# “I managed to stand on my own. I saved my baby’s life.”: qualitative analysis of birth experiences from women living with HIV in Cape Town, South Africa

**DOI:** 10.1186/s12978-024-01881-3

**Published:** 2024-10-08

**Authors:** Alison Z. Weber, Destry Jensen, Kira DiClemente-Bosco, Nokwazi Tsawe, Lucia Knight, Landon Myer, Jennifer A. Pellowski

**Affiliations:** 1 Department of Behavioral and Social Sciences, Brown University School of Public Health, 121 South Main Street, Box G-S121-3, Providence, RI 02912 USA; 2 Institute of Public Health and Medicine, Center for Dissemination and Implementation Science, Northwestern University Feinberg School of Medicine, Evanston, IL USA; 3 Division of Epidemiology and Biostatistics, School of Public Health, University of Cape Town, Cape Town, South Africa; 4 Division of Social and Behavioural Sciences, School of Public Health, Faculty of Health Sciences, University of Cape Town, Cape Town, South Africa; 5 School of Public Health, Community and Health Sciences, University of the Western Cape, Bellville, South Africa; 6 International Health Institute, Brown University School of Public Health, Providence, RI USA

**Keywords:** Pregnancy, Birth experiences, HIV, Obstetric violence, South Africa, Structural vulnerability, Respectful maternity care

## Abstract

**Background:**

There is growing recognition of obstetric violence in health facilities across the globe. With nearly one in three pregnant women living with HIV in South Africa, it is important to consider the influence of HIV status on birth experiences, including potential experience of obstetric violence as defined by the Respectful Maternity Care Charter. This qualitative analysis aims to understand the factors that shape birth experiences of women living with HIV, including experiences at the nexus of HIV status and obstetric violence, and how women react to these factors.

**Methods:**

Data were collected in a Midwife Obstetric Unit in Gugulethu, Cape Town, South Africa, through 26 in-depth interviews with women living with HIV at 6–8 weeks postpartum. Interviews included questions about labor and early motherhood, ART adherence, and social contexts. We combined template style thematic analysis and matrix analysis to refine themes and subthemes.

**Results:**

Participants described a range of social and structural factors they felt influenced their birth experiences, including lack of resources and institutional policies. While some participants described positive interactions with healthcare providers, several described instances of obstetric violence, including being ignored and denied care. Nearly all participants, even those who described instances of obstetric violence, described themselves as strong and independent during their birth experiences. Participants reacted to birth experiences by shifting their family planning intentions, forming attitudes toward the health facility, and taking responsibility for their own and their babies’ safety during birth.

**Conclusions:**

Narratives of negative birth experiences among some women living with HIV reveal a constellation of factors that produce obstetric violence, reflective of social hierarchies and networks of power relations. Participant accounts indicate the need for future research explicitly examining how structural vulnerability shapes birth experiences for women living with HIV in South Africa. These birth stories should also guide future intervention and advocacy work, sparking initiatives to advance compassionate maternity care across health facilities in South Africa, with relevance for other comparable settings.

**Supplementary Information:**

The online version contains supplementary material available at 10.1186/s12978-024-01881-3.

## Plain language summary

Mistreatment of women during childbirth is a global concern, with known negative impacts on the birthing person and newborn. Women living with HIV are at risk for mistreatment in clinical settings due to persistent stigma and negative perceptions about HIV. Women living with HIV may be further at risk for mistreatment during labor and delivery based on stigma related to HIV status. This qualitative data analysis aims to understand the factors that shape birth experiences of women living with HIV, and how women react to those factors. Data were collected in a Midwife Obstetric Unit in Gugulethu, Cape Town, South Africa, through 26 interviews with women living with HIV at 6-8 weeks postpartum. Interviews included questions about labor and early motherhood experiences. We used a combination of qualitative data analysis techniques to understand and organize participant experiences. While some participants described positive interactions with healthcare providers, several described mistreatment including being ignored, disrespected, denied care, and denied informed consent. Participants also said that lack of healthcare facility resources and infrastructure issues influenced their birth experiences. Nearly all participants, even those who described mistreatment during childbirth, described themselves as strong and independent. These birth stories should guide future research and advocacy in South Africa.

**Supplementary Information:**

The online version contains supplementary material available at 10.1186/s12978-024-01881-3.

## Introduction

Obstetric violence, defined as violence against pregnant or birthing people (hereafter referred to as “women”) during the provision of healthcare, includes a range of harms including neglect, lack of informed consent, and physical harm [1]. There is growing recognition of neglectful, abusive, and disrespectful treatment of women during childbirth in health facilities across the globe, occurring at the level of interaction between the woman and provider, as well as through systemic failures at the health facility and health system levels [2–4]. There are increasing calls for work to define respectful maternity care and acknowledge the human rights of women and newborns. The Respectful Maternity Care Charter (RMCC) [5] from the White Ribbon Alliance defines ten universal rights guiding interactions between women and healthcare providers, including the right to freedom from harm and ill-treatment, the right to informed consent, and the right to be treated with respect and dignity. Advancing respectful maternity care is also aligned with the United Nations’ Sustainable Development Goals, such as goals 3 (good health and well-being), 5 (gender inequality), and 5.2 (eliminating violence against women and girls) [6].

In South Africa, efforts to prevent obstetric violence and uphold the universal rights ascribed by the RMCC are subject to various social and structural pressures, including systematic inequality, colonial legacy, and health system policies and resources [1, 7–10]. Drivers of obstetric violence are complex and intertwined, including: widespread poverty and inequality, medicalization of birth, healthcare environment resources, restrictive healthcare policies, and power dynamics between healthcare providers and patients [7].

Due to the high prevalence of Human Immunodeficiency Virus (HIV) in South Africa, with approximately one in three pregnant women living with HIV [11], it is important to consider the potential influence of HIV status in the context of birth experiences. Some studies suggest that women living with HIV in South Africa face increased stigma from healthcare providers due to their HIV status, causing negative experiences within healthcare settings [12–16]. Birthing women living with HIV report discrimination during birth, while midwives describe feeling anger towards childbearing women with HIV [7]. This evidence points to violations of both the RMCC and South Africa’s Final Constitution, which provides constitutional protections for pregnant women and those living with HIV to live and receive healthcare without fear of discrimination or violence [17].

Despite the potentially complex interplay between obstetric violence and HIV status, there is a limited body of research around birthing experiences, and, to our knowledge, there are no studies that examine birthing experiences of women living with HIV in public health care facilities in South Africa. To address the current knowledge gaps, this qualitative analysis aims to understand the factors that shape the birthing experiences of women living with HIV in public birthing facilities in Cape Town, South Africa, and the ways in which women react to these factors.

## Methods

### Data collection

This study was conducted in the midwife obstetric unit (MOU) of a community healthcare clinic in Gugulethu, Cape Town, South Africa. The clinic primarily serves isiXhosa-speaking Black South African women. Gugulethu is a former African township, which experienced enforced residence of Black Africans under the apartheid government [18]. Present day residents of Gugulethu live with the effects of historic marginalization, including poverty, unemployment, and limited access to structural resources such as water, sanitation and electricity [19]. Compared to other provinces in South Africa, Cape Town has stronger health infrastructure and serves as a hub for HIV research and knowledge, setting up clinics and healthcare services with some capacity for improvements. However, efforts are still needed to expand these resources to areas with they are most critical, particularly within the former townships like that of Gugulethu, where residents often struggle with meeting basic needs. In Gugulethu, uncomplicated and low-risk deliveries occur at MOUs while higher risk deliveries occur at secondary or tertiary hospital facilities. The MOU is staffed by midwives and auxiliary nurses and refers births requiring intervention to higher-level facilities where patients can receive care from a doctor.

These data were collected within a larger longitudinal qualitative study to understand suboptimal antiretroviral therapy (ART) adherence across the peripartum transition [20, 21]. Study recruitment, data collection materials, and procedures were approved by the University of Cape Town’s Faculty of Health Sciences’ Human Research Ethics Committee as well as the Brown University Institutional Review Board (IRB) through an IRB Authorization Agreement. The study follows women from pregnancy through their first year postpartum, with in-depth interviews at four time points (32–35 weeks pregnant, and 6–8 weeks, 4–6 months, and 9–12 months postpartum). Female research staff recruited clinic attendees for study participation. Inclusion criteria were: (1) 18 years of age or older; (2) 32–35 weeks pregnant; (3) living with HIV; (4) currently prescribed ART; and (5) English or isiXhosa speaking. Potential participants were excluded if they: (1) had a high-risk pregnancy for reasons other than HIV status (i.e., preeclampsia, hypertension); (2) were currently enrolled in another ART adherence-related study; and/or (3) were unable to provide informed consent. Participants with a range of lived experience were recruited according to a non-probability, purposive sampling matrix along key axes of diversity: age, parity, and educational attainment. All participants provided informed consent prior to enrollment. All participants who were eligible agreed to participate in the longitudinal qualitative study (n = 30); however, four participants could not be reached to complete the 6–8 week postpartum interview.

The present analysis focuses on individual semi-structured interviews conducted with twenty-six women living with HIV between June and October 2018. Participants were 6–8 weeks postpartum. Interviews lasted approximately 60 min and followed a topic guide that included questions about birth and early motherhood experiences, access to healthcare, medication adherence, family and community factors, and cultural beliefs. Though this research was conducted with women living with HIV, the interview guide did not specifically probe the influence of their HIV status on their birthing experiences. All interviews were conducted in a private clinic room. Interviews were audio-recorded, transcribed, and translated into English by bilingual study staff. Interview staff also documented field notes during each interview. The study team met regularly to discuss and evaluate data saturation. Participants did not provide feedback to these findings due to the length of time between interview conclusion and undertaking this secondary data analysis.

### Data analysis

Data were organized using NVivo Version 20.5 (©QSR International, 1999–2018). After reading through the complete set of Time 2 transcripts (6–8 weeks postpartum), the first and second authors identified richness in participants’ descriptions of their birth experiences, with preliminary patterns around social and structural factors perceived to negatively impact birth experiences. Given the available data and preliminary findings, the authors used the ‘follow the template’ analysis approach defined by Brooks et al. [22] Template style thematic analysis (TSTA) uses a hierarchical coding structure, which is advantageous for our team-based coding and analysis, while allowing flexibility to adapt and refine the coding template over time using a combination of deductive and inductive coding, with iterative revision of the coding template [22] This analysis technique was supplemented with matrix analysis [23] to refine themes and subthemes. Matrix analysis enabled both cross-case and within-case analysis. The combination of analysis techniques allowed us to portray the depth of birth experiences shared by participants.

Two coders (AZW, DJ) developed an a priori codebook and hierarchical coding template guided by our research questions. The coding template included deductive codes reflecting factors that may shape birthing experiences of women living with HIV, including aspects of agency, discrimination, and the healthcare environment. Deductive codes were informed by current literature around birth experiences. Our analysis team reviewed this codebook and developed preliminary inductive codes based on our initial reading of the transcripts. For example, with in the “Participant’s Labor and Delivery Experience” parent code, the team developed inductive codes such as physical sensations, knowledge, and transportation.

After coming to consensus on the initial codebook, two coders from our team (AZW, DJ) independently hand coded two transcripts. The initial two transcripts were selected based on richness in the participants’ birth experience, to allow the coders to focus expanding the inductive codes in the coding template. The two coders met with the third author (KDB) to resolve discrepancies in how the codes were interpreted and applied. The third author attended these meetings to provide high-level suggestions for codebook revisions. After double-coding six transcripts with consensus, coders independently coded four transcripts and double-coded two transcripts. The full coding team then met again to discuss changes in the coding template and codebook. This process was repeated until all transcripts were coded.

For this manuscript, coded text fragments were clustered into themes and sub-themes, with relevant quotes organized within a data analysis matrix [23]. The matrix analysis approach allowed for further refinement and comparison of themes and sub-themes across participants. The team engaged in regular meetings to facilitate identification and refinement of themes. Preliminary findings were reviewed and presented to the full authorship team, sparking additional data review and further refinement. In the process of theme refinement, additional within-case analysis was undertaken for participants reporting experiences of obstetric violence. Supplementing the across-case analysis with within-case analysis better portrays the experiences shared with our research team, and is consistent with established qualitative data analysis approaches [24]. Preliminary themes were reviewed and presented to the full authorship team, sparking additional data review and further refinement of the themes. Manuscript preparation followed COREQ guidelines (Table S1; [25]).

## Results

All participants in this study (n = 26) were Black South African women living with HIV, who were 6–8 weeks postpartum at the time of the interviews. Participants were an average of 28 years old, with the majority being single (never married) homemakers, with a household income less than R1,000 per month (about $68 USD in 2018; Table 1). All participants birthed in a public healthcare facility (public clinic or hospital). Most were multiparous and had a vaginal delivery.

**Table 1:** Participant demographics at 6–8 weeks postpartum (N = 26)

	Mean ± SD
Age (years)	28 ± 6.4
	% (n)
Race	
Black South African	100 (26)
Marital status	
Single (Never married)	62 (16)
Not married (in marriage-like relationship)	19 (5)
Married	19 (5)
Employment status	
Currently employed	0 (0)
Self-employed	0 (0)
Looking for work/unemployed	4 (1)
Temporarily laid off	12 (3)
Homemaker	81 (21)
Student	4 (1)
Other	0 (0)
Average monthly household income	
< R1,000 per month	62 (16)
R1,000—R5,000 Per month	19 (5)
R5,000—R10,000 Per month	19 (5)
> R10,000 Per month	0 (0)
Number of biological children (including most recent birth)	
1	27 (7)
2	50 (13)
3	23 (6)
Delivery setting	
Public clinic	54 (14)
Public hospital	46 (12)
Birth type	
Vaginal	73 (19)
Cesarean	27 (7)

Participants described a range of social and structural factors they felt influenced their birth experiences, including lack of resources and institutional policies. While many participants described positive interactions with healthcare providers, several described instances of obstetric violence. Nearly all participants –even those who described instances of obstetric violence—described themselves as strong and independent during their birth experiences.

### Theme 1: participants described social and structural factors underlying their birth experiences

Participants described a range of social factors that they perceived to influence their birth experience, including institutional resources and policies, and structural resources. Though not detailed here, participants highlighted receiving social support from friends and family, including financial support for travel to the hospital, transportation, and emotional support. Participants described mixed experiences of social support from partners [26]. While some described partners as “not supportive” (PID 101, hospital), or unreliable, others said their partners were “supportive all the time” (PID 102, hospital). A few participants described social norms and beliefs shaping their birth experiences, such as the belief that birthing a baby boy is more painful than a girl.

#### Institutional resources

Participants noted that health facilities’ access to resources—framed here as institutional resources—influenced to their birth experiences. Some perceived that access to resources such as heartbeat monitor belts, cesarean section, and induction services gave them the impression that they were getting high quality care at advanced care hospitals, where participants with complex births or complications delivered their babies. One participant talked about access to free services, explaining:

*"So here everything is for free. Just buy things for your baby. And you go and deliver well, you go back home without paying anything, medication is free… [This] make[s] me feel comfortable. Very comfortable."* (PID 125, clinic)

Others described lack of resources – particularly at MOUs – such as having to wait for a bed or ambulance to become available, as being disruptive or distressing. As one participant shared, “*I was told to wait because the beds were occupied. I was then admitted later on.”* (PID 111, clinic) Despite being denied a service that should be available, this participant did not express any concerns about the delay in being admitted, or any impact on her birth experience. In contrast, another participant described lack of institutional resources as a key factor in her negative experience (see Table 3 for more information about this participant’s experience). Briefly, the participant explained that “*because there were no ambulances available…my baby had to leave first, and I followed a bit later*” (PID 123, clinic). The participant went on to describe perceived lack of resources at the hospital where her baby was being treated. Though her baby was in the ICU for several days, the participant was “*informed that there were no beds available for me, I slept in the passages for days*.” When reflecting on these experiences, the participant described “*feeling very down*” and was referred by study staff to speak with social workers.

#### Institutional policies

Participants described institutional policies as immutable. One participant who experienced obstetric violence during a traumatic birth referred to an MOU discharge policy, saying “*you have to be discharged after six hours anyway”* (PID 103, clinic; Table 2). This participant described feeling distressed by the short discharge period, as there was *“no one to fetch me*” when the six hours passed. She said that she “*took a bath and changed clothes*” before getting a taxi home. This participant described conflicting feelings of being rushed out and simultaneously wanting to leave so she did not have to see the nurses anymore. In contrast, one participant described feeling frustrated that she had to stay overnight in the MOU, as she preferred to go home more quickly. She said *“…because (the birth was) late at night I will be forced to sleep in the clinic just for the night. I was very frustrated*.” (PID 108, clinic) Other participants blamed the health facilities for lack of support while giving birth, due to perceived policy that partners and family members are not allowed to be with the patient during delivery, stating “*there was no one [present] to give me support system*” (PID 101, hospital).

**Table 2:** PID 103—clinic birth—case analysis

Birth narrative	Respectful maternity care charter violations
In describing her birth experience, the participant provides a detailed account of her baby’s birth. At the beginning of her labor, she describes the nurses being “*fast asleep.”* As her labor progressed, she could feel the baby was coming, and called for the nurse. When attending to her, the nurse “*told me there was no baby coming and instructed me to step down from the bed (to keep walking).”* Following the nurse’s instructions, the participant stepped off the bed, and leaked mucous or fluids on the floor. The participant describes the ensuing interaction with the nurse as follows: *“The nurse who instructed me to step down from the bed shouted at me wanted the reason for giving birth (likely mucous or fluids) on the floor. I replied to her that she is the one who instructed me to step down from the bed and I had to follow her instructions as I was afraid of her. The floor was dirty, and she gave me something to clean the floor. […] She said, ‘I can’t work on a dirty area; you must clean the floor because there are no cleaners over the night.’ I started to clean, and I could still feel that my baby was coming.”* The participant explained that her baby was not born yet when she was cleaning the floor, but “*her head was out already.”* She described being afraid of the nurse and feeling that she had to follow the nurse’s instructions, even though the baby was coming. She goes on to say: *“I started by cleaning where the nurse was going to walk pass[ed] and my water broke (likely mucous or fluids) while I was busy cleaning.”* The participant says, “*I* *ended up giving birth to my baby while I was standing on the floor.”* She describes the birth as follows: “…*a nurse instructed me to catch my baby. She [the nurse] said, ‘Who has to catch your baby if you don’t?*’ […] *The ward was dirty by then. She said, ‘What must I do and how could I attend to you when the ward is dirty like this?’ I said, ‘I told you from the beginning that my baby is coming; you were supposed to attend to me long time ago’ She said, ‘Stay there; I will take your baby and I will leave you dirty like that until I have time to attend to you’. I ended up asking what I have done to her; maybe she knows me, and I don’t know her. She said, ‘You have never done anything to me; do you think I treat you different from other patients?’ I said ‘It is obvious that you treat us all like this’. That’s how it went.”* Reflecting on her experience, this participant states: *“…I heard that the staff in there [at the healthcare facility] mistreats their patients. I never took what I heard serious; I wanted to experience it. I believed what I heard after I had such a bad experience.”* She went on to say: “*[I will] never come to [hospital name] again*” after having negative experiences. “*I might as well have done everything at home because there was no need for me to go to the hospital if I could do it on my own whereas there are people who were hired to take care of us.”*	Harm and ill-treatment: The participant describes receiving no help or assistance when she felt her baby was coming, or when her baby was crowning (“*her head was out already”*) The participant describes being implicitly threatened, in that she must clean the floor to access care from the nurse. The participant describes the fear she felt when she decided to follow the nurse’s instructions When her baby was born, the participant had to catch her own baby to prevent the baby from falling on the floor, despite the presence of the nurse
	Information and informed consent: When describing her child being removed from her, the participant does not indicate that she consented to this procedure. The participant described the nurse as forcefully removing her baby and leaving the participant dirty and unattended
	Dignity and respect: The participant describes several instances of disrespect, including being ignored when she tells the nurse her baby is coming, shouted at for leaking fluids on the floor, and being told to clean the floor while in labor
	Equitable care: During her interaction, the participant describes asking the nurse why she is being treated so poorly. According to the participant, the nurse says that she treats all patients the same, in that all birthing women are treated disrespectfully by the nurse
	Access to healthcare: The participant is denied access to care due to the “dirty area,” and is seemingly punished for leaking fluids during her labor Ultimately, the participant describes a desire to avoid the hospital in the future, because of the poor level of care provided during her birth
	Child separation The participant describes the nurse removing her baby while she is distressed, causing the participant to ask why the nurse is treating her so badly. As the participant describes it, the baby was removed without consent

#### Structural resources

Participants described how structural resources, particularly public transportation, influenced their birth experiences. Participants described paying for taxis, sometimes by borrowing money, or calling an ambulance to travel for their births. One participant, who used an ambulance to get to the health facility, said “*we called the ambulance because we couldn’t afford to hire [a] car again.*” (PID 107, clinic). The participant described the ambulance being *“escorted (into the community) by the police force,”* highlighting the need for escort due to high levels of crime and instances of ambulances being targeted in criminal acts [27]. Another participant described having to wait to go to the hospital, because “*there was no transport to take me to the hospital at that time (3am)”* (PID 119 hospital). She and her husband waited until the taxi stand opened to go to the hospital. Overall, participants indicated that lack of structural resources made their birth experiences more difficult. Alongside these factors, participants also discussed their positive and negative birth experiences as related to interpersonal dynamics with healthcare providers.

### Theme 2: Participants described both positive and negative experiences, with experiences of obstetric violence aligned with Respectful Maternity Care Charter universal rights violations

#### Positive experiences with healthcare providers

Some participants described positive, caring, and informative interactions with healthcare providers. One participant (PID 108) appreciated the care a nurse demonstrated when washing her newborn girl and providing instruction on skin-to-skin bonding between the mother and her child (PID 108, clinic). Another participant said, “*I was treated very well I don’t want to lie, because they were checking up on me and my baby to see if we are still well.”* (PID 110, hospital). Others said the nurses were “*very nice*” (PID 118, clinic), and “*well trained”* (PID 125, clinic). Participants also described getting education and advice from nurses, saying “*they taught me on how to breastfeed*” (PID 120, hospital), and “*they also taught me how to give my baby her [prophylactic] medication*” (PID 130, hospital).

Some participants described receiving advanced care for complex or emergency situations. One participant described having a cesarean section due to her high viral load:

*"I gave birth through C-Section due to my viral load which was high. Healthcare providers had to save my baby from dying and they explained to me why I had to give birth through C-section."* (PID 110, hospital)

This participant explained that the cesarean was planned during antenatal care visits to avoid potential HIV transmission to the child during labor and delivery, and described understanding the need for the procedure, and feeling informed about the procedure. Another participant described an emergency delivery, during which her baby’s arm was broken. She expressed mixed feelings about this experience, ranging from acceptance to frustration. She described the injury as follows:

“Participant: Baby was not coming out... […] I was in labour pains, and she was not coming out; it was only the head that was out, and the shoulders were still inside the womb. They had to pull her out. […] they informed me immediately [about the accident]. […] It was obvious because her hand turned blue. They found out immediately, but they scheduled her X-Ray appointment for the following day.* […] They informed me the day they were discharging me that they will schedule an appointment for me to go to [advanced care hospital].” (PID 102, hospital).

*Underlining added for emphasis.

While the participant described feeling distressed about her baby’s injury, she did not express negative feelings toward the healthcare providers. Instead, she described being informed quickly about the complication, and her baby being transferred to an advanced care hospital to receive appropriate treatment.

#### Experiences of obstetric violence during labor and delivery

Alongside descriptions of supportive birth experiences, even during distressing circumstances, participants also detailed negative interactions with healthcare providers. Another participant described lack of information and informed consent, explaining that she was not aware she was going to give birth through a cesarean section:

*“I never wanted it (cesarean section) either. But they said the baby’s head circumference was too big. […] They never told me anything, they just sent me to the theatre because they didn’t want to risk and keep me waiting while I had already waited for a very long time.”* (PID 126, hospital)

The same participant later described being concerned when a nurse changed her urinal catheter, saying *“I was very shocked thinking I was harmed during the operation.*” (PID 126, hospital).

Three participants provided in-depth narratives that meet criteria of obstetric violence, with harms experienced across the spectrum of rights detailed in the Respectful Maternity Care Charter. One participant described being coerced into cleaning her birth fluids off the floor while her baby was crowning and having to catch her own baby (Table 2).

This participant described harms ranging from ill-treatment, lack of informed consent, disrespect, inequitable care, denied access to care, and non-consensual child separation (Table 2).

Similarly, another participant attributed her child’s birth injury to perceived negligence (Table 3).

**Table 3:** PID 123—clinic birth—case analysis

Birth narrative	Respectful maternity care charter violations
The participant describes her birth as follows: *“I stood up and I felt something coming out of my vagina…it was the head of the baby. I screamed immediately shouting and calling nurses. The other nurse responded by saying “we are not your kids come to us”. I told them that I cannot move or even raise my leg because I was afraid the baby might fall, they ignored me and continue with their conversation. I had to drag my feet to the bed, and I could feel the head of the baby was stuck in the vaginal opening. When I get to the bed, they said I should get off that bed and go to the other bed. I had to drag my feet getting to the other bed….it was this time that they realized that this was something serious they came (nurses) and assisted me to deliver. I just pushed three times and the baby came out.”* The participant goes on to describe harm to her baby that she believes is due to negligence from the nurses: *“What was shocking is that my baby didn’t cry and the head of my baby including the face turned purple because the blood was not circulating while the head was stuck in the vaginal opening, she had internal bleeding even the (baby’s) mouth was purple. […] This is a very serious case of negligence because it is not as if the nurses were busy with something else, but they were just seating on the table chatting. It was very serious, and they even said themselves that I almost lost my baby.”* The participant’s baby was transferred to an advanced care hospital without her, “*because there were no ambulances available…my baby had to leave first, and I followed a bit later.”* This participant went on to describe continued lack of resources after arriving at the hospital ICU where her baby was being treated. She said, “*When we got there, they [medical staff] were busy attending to her….it was very bad, as you can see the sores in her nose [from] the gastronomy tubes.”* Though her baby was in the ICU for several days, the participant was “*informed that there were no beds available for me, I slept in the passages for days.”* The participant goes on to describe her response to this incident. She states that she was “*feeling very down, in fact I was feeling down for several days.*” The participant says, “*It was after five days that I hold her (her baby) in my hands for the first time.”* She goes on to say “*(crying) I got better and better when I saw the condition of my baby was improving.”* The participant was referred by study staff to speak with social workers	Harm and ill-treatment: The participant describes receiving no help or assistance when she felt her baby was coming, or when she felt her baby was stuck The participant also describes being treated as though she does not deserve care, when the nurse tells her “*We are not your kids come to us*.” The participant perceives that her baby’s injury and resulting ICU stay were due to negligence from the nursing staff
	Information and informed consent: The participant describes confusion when trying to follow directions from the nurses, regarding which bed the participant should be on for birth
	Dignity and respect: The participant describes being treated disrespectfully, with nurses ignoring her calls for help and verbally dismissing her The participant describes the nurses socializing with each other rather than providing her with requested care
	Access to healthcare: The participant describes being denied necessary medical care when the baby was stuck. She further describes lack of resources to emergency services, such as an ambulance for transportation, and facilities for her to stay with her baby in the ICU
	Child separation: The participant describes her baby being separated from her due to the lack of ambulances to transfer them to the advanced care facility together. She does not describe understanding or consent around separation from her child. She goes on to describe sleeping in hallways to stay with her baby in the ICU. The participant describes being distressed at being separated from her baby during transfer, and during the ICU stay

This participant described harms across similar domains, including ill-treatment, lack of information, being treated disrespectfully, and being denied access to care during labor.

The final participant highlighted being ignored by nurses after repeatedly asking for help, ultimately forcing the participant to catch her own baby (Table 4).

**Table 4:** PID 127—clinic birth—case analysis

Birth narrative	Respectful maternity care charter violations
The participant describes the support from the nurses as inattentive, saying the “*nurses [left] us alone in the labour ward to watch TV.”* She goes on to describe her delivery: *“My water broke when I was lying on the bed in the labour ward then I reported to them. They sent one of the student nurses to let me know that I was making noise. […] They said I must walk up and down the labour ward. They never bothered to check up on me after that. […] I decided to go straight to them to show them how serious I was. Water was running down on my legs, but they instructed me to walk up and down. […] I felt like pushing while I was standing in-front of the nurses. I pushed and my baby came; fortunately, I managed to hold him with my gown. […] I first saw his head with hair and then arms followed while I was still standing. I had to hold him with my gown until I got into bed.”* The participant goes on to describe how she felt about her birth experience. She says, “*I was angry, and I started to hate this hospital… I heard [the same] from other people until I went through the same situation.”* Though the participant describes being proud of saving her baby, she also alludes to the trauma she experienced during her delivery: *“If I was someone else; I would wait for nurse’s help, but I managed to stand on my own. I saved my baby’s life.”*	Harm and ill-treatment: Like other participants, this participant describes being ignored by nurses when she calls for help, including when her baby is born while she is standing in front of the nurses
	Dignity and respect: In response to calls for help, a nurse is sent to tell the participant she is making too much noise The participant describes asking for help multiple times, with nurses dismissing her concerns each time
	Access to healthcare: The participant describes care being withheld by the nurses. When asking for help, the nurses tell her to walk and do not check on her again. The participant ultimately approaches the nurses and delivers the baby while standing in front of the nurses; catching her own baby instead of getting help from the nurses

This participant described harms across several domains, including ill-treatment, being treated disrespectfully, and being denied access to care during labor.

The participant experiences included in these case studies highlight interpersonal dynamics between birthing women and healthcare workers as key factors driving birth experiences. However, all participants also discussed their strength and independence as birthing women.

### Theme 3: participant (re)actions highlight strength and independence

Despite the challenges participants described when telling their birth stories, nearly all participants highlighted their own strength and independence. When reflecting on their birth experiences, participants described feeling strong and independent, shifting family planning intentions, and forming attitudes towards the health providers and facilities.

#### Feeling strong and independent

Participants described their independence, strength, and ability to take care of themselves and their babies during birth. In some cases, participants said they had to be strong and independent because “*there’s no other way”* (PID 101, hospital), referring to the lack of labor and delivery support from nurses in health facilities. One participant (PID 127, clinic; Table 4) described feeling strong after protecting her baby from injury during a traumatic birth, saying:

“If I was someone else; I would wait for nurse’s help, but I managed to stand on my own. I saved my baby’s life.*” (PID 127, clinic)

*Underlining added for emphasis.

Several participants described feeling strong *because* they gave birth. One said,

“*I felt strong, I was a mother who managed to survive the challenges [during delivery]. I don’t think there is any woman who would carry a baby in her tummy for 9 months and feel less of herself. The fact that I managed to deliver my baby made me proud.”* (PID 128, hospital)

One participant described feeling strong after birth, saying: “*The fact that I coped well to give birth after I suffered from lot of labour pains shows that I am a strong woman.”* (PID 102, hospital) Another said trusting herself helped with labour: “*I had to be strong in difficult times (of labor). […] Trusting in myself has helped me a lot.”* (PID 115, clinic) One described the feeling powerful after healing from a cesarean section, saying “*I felt powerful because I was able to survive the pains I went through.”* (PID 114, hospital) Participants went on to describe how giving birth motivated them to continue caring for themselves and adhering to HIV treatment regimens. One participant said “*Giving birth to a healthy baby has motivated me to carry on with everything. My kids are the reason I am alive*.” (PID 121, clinic).

#### Future family planning

Several participants described shifting their future family planning intentions based on their birth experiences. One said, “*I told myself that this baby is my first and last born, I will never have other children ever again.”* (PID 129, clinic) This participant explained that her decision was driven by having “*too much pains”* during the birth. Another said, “*I told myself that I will never have other children again […] I said I want two children, but [….] experience I received from labor ward has changed my minds.”* (PID 130, hospital) Another said, “*I just told myself that I will never have another baby after what I have gone through during this pregnancy.”* (PID 114, hospital) Several participants said that they did not want to have more children because of the negative experiences they had during pregnancy and labor.

#### Forming attitudes toward the health facility

Participants described a range of feelings about health providers and facilities after their birth experiences. Participants who formed positive opinions and attitudes toward the healthcare providers and facilities described trusting the nurses and having good birth experiences. These participants described the healthcare providers as “nice” and said treatment from nurses was “very good”. One participant said: “*I like to give birth here in (the) MOU.”* (PID 107, clinic) Most participants with positive opinions had low risk, uncomplicated births at the MOU or a planned cesarean.

In contrast, participants who had complicated or traumatic births tended to describe negative opinions of the healthcare providers and facilities. One participant asserted her right to supportive healthcare and power in choosing healthcare facilities, saying “*[I will] never come to [hospital name] again*” (PID 103, clinic birth; Table 2) after her traumatic experience. After a similarly traumatic birth, PID 127 (Table 4) said “*I was angry, and I started to hate this hospital.”*

These responses showcase the ways in which women reacted to their birth experiences, feeling strong and independent because of—or despite—their birth experiences, shifting their family planning intentions, and forming attitudes about healthcare providers.

## Discussion

A woman’s birthing experience is an important, deeply personal life event that should be treated with the utmost care and consideration. Findings suggest that while many participants had positive birthing experiences, others shared negative experiences aligned with Respectful Maternity Care Charter (RMCC) violations. Various structural and social factors influenced birth experiences, including institutional resources and policies, and structural resources. Nearly all participants highlighted their strength and independence when reflecting on their birth experiences. 

The experiences and priorities encountered in this study are consistent with current literature. Although existing research on the topic is limited, narratives of disrespectful labor and delivery care found in this study have previously been reported throughout South Africa [4, 8, 9, 28]. Findings of negative birthing experiences in this study also aligned with RMCC violations [5]. Participants felt that factors such as lack of transportation and financial constraints negatively impacted healthcare access for pregnant and postpartum women, consistent with existing literature [29]. Further, they felt that rigid policies impacted their birthing experiences, consistent with previous studies in South Africa [28]. Institutional policies also contributed to participants feeling a lack of support, due to their perception that the healthcare facility rules prohibited family and friends from being present during the birth. Similar reports of denied companionship during labor has been previously shown, despite evidence linking birth support to positive birth outcomes [30, 31].

Participant experiences speak to the responsibility forced on birthing individuals because of broader system failures, including lack of support from nursing staff, limited resources, and experience of disrespect and mistreatment during birth. Narratives from participants describing obstetric violence illustrate the social hierarchy between a birthing person and healthcare providers, in which healthcare providers hold and use power to control birthing women. The impact of social hierarchy on patient-provider interactions in South Africa has been previously demonstrated [3, 9, 32]. In this study, instances such as a nurse responding to a participant’s call for help by saying “*We are not your kids come to us,*” or forcing a laboring woman to clean up her own birth fluids before getting help illustrate the violence perpetrated against birthing women within a complex network of power relations. This instance of disrespectful care can be viewed as a nurse’s effort to control the birthing person’s body in order to maintain her status as more powerful than the birthing person and maintain her power and control during the birth [6]. In another example, a nurse was described as punishing a participant, when she took a participant’s baby and said she would leave the participant ‘*dirty like that.’* This interaction can be viewed as another example of a nurse exerting control over both the newborn and birthing person’s bodies, exerting her power to punish the woman by denying her care. Prior studies have also reported healthcare providers attempting to control a birthing woman’s body through physical or verbal means [4, 8, 9, 28, 32]. Ultimately, a constellation of factors produces disrespectful care via efforts to control knowledge and control bodies, keeping power and control in the hands of the healthcare providers [7].

### Limitations and strengths

The main limitation to this retrospective analysis is that the interviews did not explicitly ask participants to describe their perceptions about how their HIV status may have influenced their birth experiences. Therefore, the interview data does not include nuanced perspectives about how participants feel their HIV status is related to their birth experience. It is therefore unclear whether participants did not explicitly speak about the connection between their HIV status and birth experience because they were not asked, or if this connection was not meaningful to participants. Despite this limitation, this study’s findings are consistent with prior literature which documents discrimination against birthing women living with HIV in southern Africa [12–14]. The participants’ experiences of abuse and neglect are also consistent with documented challenges in the wider public healthcare system in South Africa [33]. To our knowledge, this is the first analysis of birth experiences among women living with HIV in Cape Town, South Africa. Thus, while the study did not deeply interrogate the connection between HIV status and birth experiences, the study is uniquely positioned to provide insights around birth experiences of women living with HIV in this context. A further strength is that interview participants were interviewed 6–8 weeks postpartum, minimizing recall bias during interviews for participants reflecting on their birth experiences. All participants received care from the public health care system, with some births in both clinic and hospital settings. The variation in birth settings provides information about birth experiences in different types of public health care facilities, providing additional insights related to the public healthcare system in Cape Town.

### Public health and research implications

The study findings have several implications for healthcare policy and practice in South Africa. First, these findings support calls for healthcare facilities to allow birthing people to bring a companion in with them during labor and delivery [31]. Birth companions have been shown to support laboring people by providing informational support, practical support to find laboring positions, emotional support, and advocacy for the laboring person [34]. Additionally, the current guidelines for Maternity Care in South Africa recommend that friends and family be allowed to provide companionship during labor [35]. These findings provide further evidence supporting implementation of these guidelines, particularly in cases where infrastructure modifications are required to allow privacy for birthing people and their companions [36]. The findings also indicate that many birthing women living with HIV have positive experiences with healthcare providers, suggesting that there are existing strengths within public birthing facilities in South Africa. These strengths may be able to be leveraged to diminish experiences of obstetric violence using implementation science approaches [37]. By improving access to birth companions and building on existing strengths in the public health system, experiences of obstetric violence and negative birth outcomes may decrease for birthing women living with HIV in South Africa. Given the experiences with obstetric violence recounted here, future research should also address the mental health needs of birthing women living with HIV, as well as the mental health implications of obstetric violence among this population.

This study’s findings suggest that a complex interplay between HIV status and discrimination (at the interpersonal, social, and structural levels) may produce experiences of obstetric violence. The negative birth experiences reported in this study underscore the need for future research to explore obstetric violence from a lens of structural vulnerability. A structural vulnerability framework focuses on social structures that produce and organize suffering, and moves attention away from victimizing or blaming outcomes on the basis of individual behavior [38] Bourgois et al. [39] proposed a structural vulnerability framework to understand the ways in which local hierarchies, power relationships, and societally imposed risk factors influence healthcare experiences. Their proposed framework includes domains such as risk environments, social networks, and discrimination [39]. Future research should use a framework such as this one to deeply explore the links between obstetric violence, structural vulnerability, and HIV status. For example, future work should assess whether obstetric violence experienced by women living with HIV is perceived as related to healthcare provider discrimination based on the birthing woman’s HIV status. Further, understanding whether birthing women living with HIV feel safe birthing in healthcare facilities, with exploration of what fuels feelings of being unsafe would provide information about healthcare policy and resource modifications that could improve care experiences for birthing women living with HIV. Within the structural vulnerability framework, understanding the basis of vulnerability could yield implications for integrating structural competency into healthcare education and healthcare policy.

## Conclusion

This study sought to understand the factors that shape the birthing experiences of women living with HIV in South Africa and the ways in which women react to such factors. Narratives of negative birth experiences among some women living with HIV reveal a multitude of factors that produce obstetric violence, reflective of social hierarchies and networks of power relations. Though relatively few participants described obstetric violence, these traumatic stories illustrate gaps in achieving respectful maternity care for all women, as defined under the Respectful Maternity Care Charter. Nearly all participants, despite traumatic experiences, described themselves as strong and independent during childbirth. While women’s self-reflections are largely positive, this does not absolve the health system and health providers from taking responsibility for harms and systemic failures in the South African public health system, as detailed in the RMCC. Participant accounts of social and structural factors shaping their birth experiences indicates the need for future research explicitly examining the role of structural vulnerability in shaping the birth experiences of women living with HIV. The birth stories reported here should drive future research and intervention work, and guide initiatives to advance compassionate maternity care across health facilities in South Africa, with relevance for other comparable settings.

## Supplementary Information

Supplementary material 1. Table S1: COREQ Checklist.

## Data Availability

The data and analysis codes for this study are available from the corresponding author on reasonable request.

## Abbreviations

ART: Antiretroviral therapy
HIV: Human immunodeficiency virus
IRB: Institutional review board
MOU: Midwife obstetric unit
PID: Participant ID
RMCC: Respectful maternity care charter
TSTA: Template style thematic analysis
