# Supplementary material for: “I managed to stand on my own. I saved my baby’s life.”: qualitative analysis of birth experiences from women living with HIV in Cape Town, South Africa
Source: Reprod Health. 2024 Oct 8;21:142. doi: 10.1186/s12978-024-01881-3 (PMC11462691; doi:10.1186/s12978-024-01881-3)
Supplement: Supplementary file 1 — Supplementary material 1. Table S1: COREQ Checklist. [file 12978_2024_1881_MOESM1_ESM.docx]

**Table S1: Consolidated Criteria for Reporting Qualitative Research Checklist**

| Section / topic | Item No | Checklist item | Reported on page No |
| --- | --- | --- | --- |
| **Domain 1: research team and reflexivity** | | |  |
| *Personal characteristics* | | |  |
| Interviewer | 1 | Which author/s conducted the interview or focus group? | 6 |
| Credentials | 2 | What were the researcher’s credentials? E.g., PhD, MD | 1 |
| Occupation | 3 | What was their occupation at the time of the study? | 1 |
| Gender | 4 | Was the researcher male or female? | 6 |
| Experience / training | 5 | What experience or training did the researcher have? | 1 |
| *Relationship with participants* | | |  |
| Relationship established | 6 | Was a relationship established prior to study commencement? | 5 |
| Participant knowledge of  interviewer | 7 | What did the participants know about the researcher? E.g., personal goals, reasons for doing the  research | 5 |
| Interviewer characteristics | 8 | What characteristics were reported about the interviewer/facilitator? E.g., bias, assumptions,  reasons and interests in the research topic | 1 |
| **Domain 2: study design** | | |  |
| *Theoretical framework* | | |  |
| Methodological orientation | 9 | What methodological orientation was stated to underpin the study? E.g., grounded theory,  discourse analysis, ethnography, phenomenology, content analysis | 7 |
| *Participant selection* | | |  |
| Sampling | 10 | How were participants selected? E.g., purposive, convenience, consecutive, snowball | 6 |
| Method of approach | 11 | How were participants approached? E.g., face-to-face, telephone, mail, email | 5 |
| Sample size | 12 | How many participants were in the study? | 6 |
| Non-participation | 13 | How many people refused to participate or dropped out? Reasons? | 6 |
| *Setting* |  |  |  |
| Setting of data collection | 14 | Where was the data collected? E.g., home, clinic, workplace | 6 |
| Non-participants | 15 | Was anyone else present besides the participants and researchers? | 6 |
| Description of sample | 16 | What are the important characteristics of the sample? E.g., demographic data, date | Table 1 |
| *Data collection* |  |  |  |
| Interview guide | 17 | Were questions, prompts, guides provided by the authors? Was it pilot tested? | 6 |
| Repeat interviews | 18 | Were repeat interviews carried out? If yes, how many? | 5-6 |
| Audio / visual recording | 19 | Did the research use audio or visual recording to collect the data? | 6 |
| Field notes | 20 | Were field notes made during and/or after the interview or focus group? | 6 |
| Duration | 21 | What was the duration of the interviews or focus group? | 6 |
| Data saturation | 22 | Was data saturation discussed? | 6 |
| Transcripts returned | 23 | Were transcripts returned to participants for comment and/or correction? | 6 |
| **Domain 3: analysis and findings** | | |  |
| *Data analysis* |  |  |  |
| Number of data coders | 24 | How many data coders coded the data? | 7 |
| Description of coding tree | 25 | Did authors provide a description of the coding tree? | 7 |
| Derivation of themes | 26 | Were themes identified in advance or derived from the data? | 7 |
| Software | 27 | What software, if applicable, was used to manage the data? | 6 |
| Participant checking | 28 | Did participants provide feedback on the findings? | 6 |
| *Reporting* |  |  |  |
| Quotations presented | 29 | Were participant quotations presented to illustrate the themes / findings? Was each  quotation identified? E.g., participant number | 8-13 |
| Data and findings consistent | 30 | Was there consistency between the data presented and the findings? | 8-13 |
| Clarity of major themes | 31 | Were major themes clearly presented in the findings? | 8-13 |
| Clarity of minor themes | 32 | Is there a description of diverse cases or discussion of minor themes? | 8-13 |
